# The identification and analysis of meristematic mutations within the apple tree that developed the *RubyMac* sport mutation

**DOI:** 10.1186/s12870-024-05628-x

**Published:** 2024-10-01

**Authors:** Hequan Sun, Patrick Abeli, José Antonio Campoy, Thea Rütjes, Kristin Krause, Wen-Biao Jiao, Randy Beaudry, Korbinian Schneeberger

**Affiliations:** 1 School of Automation Science and Engineering, Faculty of Electronic and Information Engineering, Xi’an Jiaotong University, Xi’an, 710049 China; 2 Faculty of Biology, LMU Munich, Großhaderner Str. 2, 82152 Planegg-Martinsried, Germany; 3 Department of Chromosome Biology, Max Planck Institute for Plant Breeding Research, Carl-Von-Linné-Weg 10, 50829 Cologne, Germany; 4 Department of Horticulture, Michigan State University, East Lansing, MI 48824 USA; 5 Institute for Plant Genetics, Heinrich Heine University Düsseldorf, University Street 1, 40225 Düsseldorf, Germany; 6 Cluster of Excellence On Plant Sciences, Heinrich-Heine University, Universitätsstraße 1, Düsseldorf, 40225 Germany; 7 Illumina Solutions Center Berlin, Berlin, Germany; 8 Key Laboratory of Horticultural Plant Biology (Ministry of Education), Huazhong Agricultural University, Wuhan, China

**Keywords:** Somatic mutations, Meristematic layers, *Malus domestica*, Genome sequencing, Assembly

## Abstract

**Background:**

Understanding the molecular basis of sport mutations in fruit trees has the potential to accelerate generation of improved cultivars.

**Results:**

For this, we analyzed the genome of the apple tree that developed the *RubyMac* phenotype through a sport mutation that led to the characteristic fruit coloring of this variety. Overall, we found 46 somatic mutations that distinguished the mutant and wild-type branches of the tree. In addition, we found 54 somatic gene conversions (i.e., loss-of-heterozygosity mutations) that also distinguished the two parts of the tree. Approximately 20% of the mutations were specific to individual cell lineages, suggesting that they originated from the corresponding meristematic layers. Interestingly, the de novo mutations were enriched for GC =  > AT transitions while the gene conversions showed the opposite bias for AT =  > GC transitions, suggesting that GC-biased gene conversions have the potential to counteract the AT-bias of de novo mutations. By comparing the gene expression patterns in fruit skins from mutant and wild-type branches, we found 56 differentially expressed genes including 18 involved in anthocyanin biosynthesis. While none of the differently expressed genes harbored a somatic mutation, we found that some of them in regions of the genome that were recently associated with natural variation in fruit coloration.

**Conclusion:**

Our analysis revealed insights in the characteristics of somatic change, which not only included de novo mutations but also gene conversions. Some of these somatic changes displayed strong candidate mutations for the change in fruit coloration in *RubyMac*.

**Supplementary Information:**

The online version contains supplementary material available at 10.1186/s12870-024-05628-x.

## Introduction

Somatic mutations are highly valuable in fruit tree breeding, as they can generate or improve agronomically important traits, and if observed in elite material, they do not even need to be introgressed to generate new cultivars. Bud sports in fruit trees are usually clonally propagated which keeps their somatic genomes intact and any derived somatic mutation can therefore also be passed on the next clonal generation [1–7].

Several important bud sport mutations in fruit trees as well as other plants have been recently reviewed by Foster and Aranzana [2]. Examples include changes in fruit coloration, which is not only an important trait to meet consumers’ preference but might also relate to chemical compounds benefiting human health [8–13]. Changes in the fruit coloration usually result from changes in the accumulation of anthocyanins, which are synthesized by enzymes regulated by *MYB* transcription factors [14–18].

Somatic mutations introduce changes in the DNA of individual cells and, in consequence, in the DNA of all cells that are derived from the mutated cells. If a somatic mutation occurs in the meristem, the mutation can be propagated into large sectors of the plants. However, in a recent report, Goel et al. illustrated that the identities of the meristematic layers remain mostly intact during organ development and that somatic mutations in the meristem (meristematic mutations) grow only into specific cell layers instead of all cells of a newly developing organ [19].

While the economic importance of bud sports is recognized, and some somatic mutations have been identified as layer-specific, the mutations underlying bud sports remain unknown. Here, we analyzed the somatic mutations within an apple tree (*Malus domestica* cultivar *McIntosh RubyMac*) that develops fruits with dark red skin in its upper (mutant) branches, while the apples in the lower (wildtype) branches remain pale red. Comparing 12 whole-genome sequencing datasets generated from three different types of tissues from four different samples of the tree, we found 100 somatic mutations including 53 that separated the mutant and wild-type branches of the tree. Unexpectedly, the mutations did not only introduce novel variation through spontaneous mutations (46), but included a similar number of loss-of-heterozygosity mutations (54), where wild-type heterozygous sites change to homozygous sites. The two types of mutations showed opposing nucleotide spectra and could be observed in different tissues suggesting that different types of mutational mechanisms act during the generation of somatic variation. Additional analysis of differentially expressed genes between the mutant and wildtype fruit peel revealed 56 genes with differential expression profiles including 18 involved in flavonoid or anthocyanin biosynthesis and regulation. While none of the differentially expressed genes was an obvious target of the somatic mutations, we identified mutations in regions of the genome that were recently associated with natural variation in skin coloration.

## Results

### Identification of meristematic mutations

**Fig. 1:**
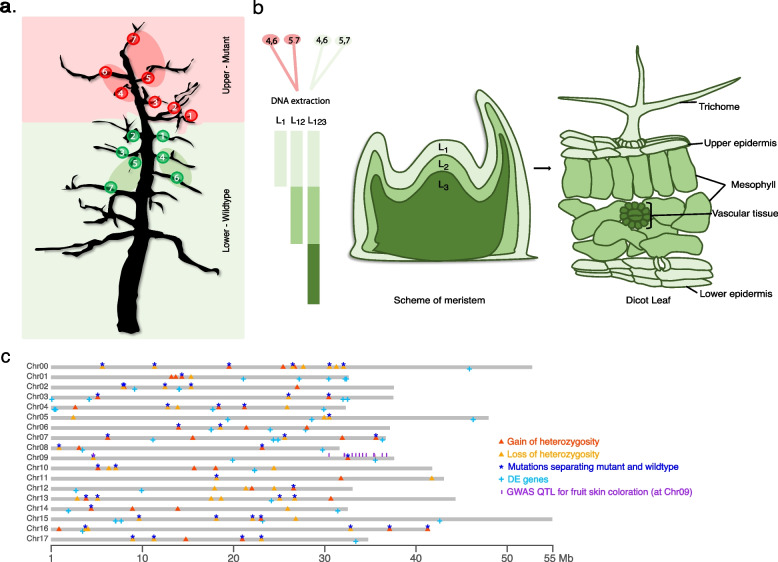
Schematics of the layer-specific whole genome sequencing based on a 3-layer meristem model, and distribution of somatic mutations along the reference genome. **a** Two-dimensional projection of the tree showing the sampling points for whole-genome as well as PCR amplicon sequencing. The pink background marks the scaffolds developing red apples, while the green background marks the scaffolds with pale red apples. The red and green eclipses show the approximated regions where the bulked leaves were selected for whole-genome sequencing. Mutant branches 1–7, wildtype branches 1–6, pooled mutant branches 4,6 and 5,7 (leaf, petiole), wildtype branches 4,6 and 5,7 (leaf, petiole) were genotyped with PCR amplicon sequencing. **b** Meristem model with three different layers and how the layers correspond to the cell layer of a leaf, i.e., *L*_*1*_: trichome and epidermis, *L*_*2*_: Mesophyll and *L*_*3*_: vascular tissue. **c**Distribution of 86 somatic mutations across the GDDH13 v1.1 genome [26]. (the sequence of the remaining 14 mutations could not be reliably mapped to the reference sequence)

The apple tree that evolved the *RubyMac* phenotype is growing in Michigan, USA (43°04′53.1"N 85°43′13.5"W). The top seven scaffolds show purple pigmentation on stamens and dark red fruits, which cannot be observed on the lower branches of the tree, where stamens remain white and fruits develop skin with pale red color (Fig. 1; Supplementary Fig. 1). The acquired phenotype is stable even after clonal propagation, and reversions have been seldomly reported. This suggested that a somatic mutation that occurred during the development of the tree could be responsible for the change in fruit coloration. As this change was present in the entire upper part of the tree including unconnected scaffolds, we assumed that this mutation occurred within the shoot apical meristem during the development of the stem and thereby separated the lower (wildtype) and upper (mutant) parts (Fig. 1a).

To identify such meristematic mutations, we sampled four different sets of leaves: two pools of leaves from connected scaffolds of the mutant part (where the selected scaffolds of the different sets were distinct) as well as two pools of leaves from connected scaffolds from the lower wildtype part of the tree (where again the scaffolds of the different sets were again distinct) (Fig. 1a). Pooling the DNA of several leaves from multiple branches dilutes the signal of somatic mutations that occurred in individual branches, while mutations that occurred in the meristem and that are propagated to the entire upper tree would be present in all leaves of a pool (in fact, would even be present in both samples of the upper part). We therefore assumed that the mutations that we identified in both samples of upper part of the tree could be candidate mutations for the sport mutation phenotype.

Following a meristematic model that supports the presence of three layers [19, 20], we extracted DNA from three different leaf sub-tissues (of each of the four pools of leaves) to enrich for cells that originated from different meristematic layers leading to a total of 12 DNA samples (Fig. 1b). The different extractions included DNA from trichomes (enriched for cells derived from layer 1 (or *L*_*1*_)), peeled leaf surface (enriched for cells derived from layer 1 and 2 (or *L*_*12*_)) and whole leaf blades (including cells derived from all three layers (or *L*_*123*_)) (Fig. 1b). We sequenced the *L*_*123*_ and *L*_*12*_ samples with 69–87 × genome coverage using Illumina 2 × 250 bp short-reads (Materials and methods). In contrast, the sequencing of the four trichome *L*_*1*_ DNA samples yielded only 4–7 × of the apple genome as there was substantial pathogen contamination in the sequencing data (Supplementary Table S1).

Using the whole genome sequencing data of one of the wildtype samples, we generated a de novo contig-level assembly of the *RubyMac* genome using *DiscovarDeNovo* [21] with a total length of 875 Mb. The assembly contained 104,393 contigs with a contig N50 of 17 kb, and a BUSCO completeness value of 91.3% at sequence level (Supplementary Table S2). Similarly, a *k*-mer based analysis showed the assembly represented 94.4% of the genome, and the base quality of the assembly reached QV67, which implies one base error per five Mbps. In addition, reference-based scaffolding of the *RubyMac* contigs allowed a comparison against the reference GDDH13 v1.1 assembly [26] revealing that genic regions were well assembled (Supplementary Fig. 32). On average, we found 1 snp per 113 bp between the two genome assemblies (Supplementary Table S8; Materials and methods). We further extracted RNA samples from the peels of both mutant and wildtype fruits (each with three replicates), from which 24–27 million Illumina 150 bp single-end reads were respectively sequenced. With the guidance of RNA-seq data, we predicted 42,981 high confidence gene models in the *RubyMac* assembly using *augustus* [22, 23] (Materials and methods).

We aligned each of the 12 sequencing read sets to the *RubyMac* assembly with an alignment rate of 92–96%, which was much higher than with any other apple reference sequence, suggesting that this was the optimal reference sequence for mutation identification in our data (Supplementary Table S1). Combing the read alignments with additional Sanger sequencing, we optimized the criteria for searching for somatic on mutations (Materials and methods; Supplementary Fig. 2–30). Overall, we were able to identify a final set of 100 single-nucleotide mutations. For further confirmation we also called mutations with the reference-based tool *MuTect* [24] and the reference-free tool *discosnp* +  + [25] and found all 100 mutations with both tools (Materials and methods). Note, the *L*_*1*_ samples were generally not used for mutation identification as the read coverage was too low for finding new mutations.

Among the 100 mutations, 53 mutations were common to both samples of the upper or common to both samples of the lower parts of the tree and therefore separated the mutant and wild-type parts, while the remaining 47 mutations were not specific to the lower or upper parts, but occurred only in one region of the tree (10 mutations), or showed a cryptic distribution across mutant and wild-type (37 mutations; Supplementary Table S3). Using the chromosome-level reference assembly of apple [26] as a guide, we found that the mutations were scattered across all chromosomes (Fig. 1c). Note, no large-scale structural variations (SVs) or transposable elements were found that would have distinguished the mutant and the wildtype branches of the tree, using SV detection tool like *CNVnator* [27] (Materials and methods).

### GC-biased gene conversions counteract AT-biased *de novo* mutations

De novo mutations introduce novel variation, which is specific to one of the two homologous chromosomes (here referred to as gain-of-heterozygosity mutations). In rare cases de novo mutations can turn a heterozygous into a homozygous allele (referred to as loss-of-heterozygosity mutations) or into a different heterozygous allele (referred to as change-of-heterozygous-allele mutations). However, the probability for loss-of-heterozygosity or change-of-heterozygous-allele mutations is very low as heterozygosity is typically low.

**Fig. 2:**
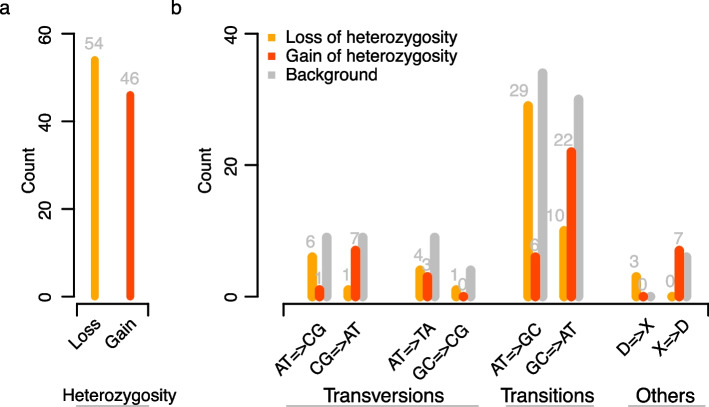
Type and spectrum of mutations. **a** 54 loss-of-heterozygosity (LOH) and 46 gain-of heterozygosity (GOH) were observed. **b** Mutational spectra of LOH and GOH mutations. The background distribution is based on germline variation (SNPs) found between both haplotypes of the apple tree

All 100 mutations could be clearly assigned to either loss or gain-of-heterozygosity mutations (Materials and methods). Surprisingly, however, loss-of-heterozygosity mutations were strikingly overrepresented (Supplementary Table S3). Overall, we found 54 loss-of-heterozygosity and 46 gain-of-heterozygosity (Fig. 2a), even though the genome-wide heterozygosity was as low as 1.2%. But despite this unexpected overrepresentation of loss-of-heterozygosity mutations, change-of-heterozygous-allele mutations were completely absent. This suggested that it was actually not de novo mutations that introduced the loss-of-heterozygosity mutations, but that they were introduced through the replacement of one allele by its homologous allele through gene conversions.

Usually, gene conversions can only be observed if the occur at heterozygous sites. Finding a similar number of mutations and gene conversions implies that gene conversions are in fact much more prevalent than mutations. This observation is similar to a finding in *Arabidopsis thaliana*, where gene conversions were found to occur orders of magnitudes more often than spontaneous mutations in a transgenerational experiment [28].

To understand more about the differences between de novo mutations and gene conversions, we analyzed their mutational spectra (Fig. 2b). In general, de novo mutations were highly enriched for GC =  > AT transitions (48%; 22 out of 46) which recapitulated the spectrum of de novo mutations that were inherited through the germline [29]. In contrast, 54% (29 out of 54) of the gene conversions were AT =  > GC transitions, while all the other types of gene conversions were underrepresented (2–19%). Interestingly, the contrasting spectra of genomic mutations and gene conversions, i.e., GC-biased gene conversions and AT-biased mutations, suggested a mechanism that could stabilize the GC content of a genome.

### Layers-specific *de novo* mutations and gene conversions

Meristematic mutations can be propagated into large parts of a plant and thereby introduce mutant sectors [30]. However, as the meristem is organized in layers [20], meristematic mutations might be propagated only into specific cell lineages of the sectors that they can be found in. Specifically, the strength of the somatic bottleneck that cells from the meristematic layers go through when initiating lateral branches could be a biological factor that affect the amount of the mutant alleles. In turn, this implies that somatic mutations could only be present in a fraction of the cells despite being present in large areas of the tree. The actual mutant allele frequency in the whole-genome sequencing data therefore depends on the fraction of cells of the mutated layer in the sequenced sample. In addition, the mutant allele frequency in the aligned reads also depends on the reference sequence, which either allows the alignment of the reads of both alleles (in the case the reference sequence is assembled as *homotig* (both alleles merged in the reference sequence assembly)) or which only allows the alignments of only one of the alleles (in the case the reference sequence is assembled as *haplotig*) in the respective region.

**Fig. 3:**
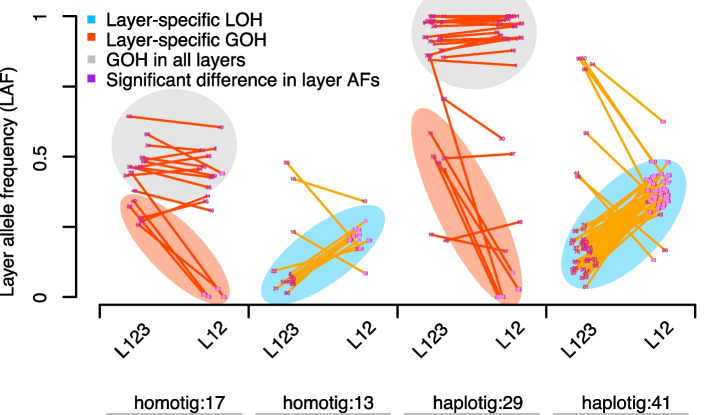
Layer-specificity of mutations. LOH and GOH occurred in three major clusters in respect to the read frequency within two different types of samples (*L*_*123*_ and *L*_*12*_ samples) indicating their occurrence in different cell layers (Material and methods). The clusters were consistently found on *haplotigs* as well as *homotigs* (where the reads of the other allele were aligned as well). The grey cluster shows de novo mutations that occur in almost all reads of the haplotype independent of the actual sample (*L*_*123*_ or *L*_*12*_). The red cluster shows de novo mutations that occurred in approximately half of the reads in the *L*_*123*_ samples but were almost entirely absent in the *L*_*12*_ samples. The blue cluster shows loss-of-heterozygosity mutations which were observed at low frequency in *L*_*123*_ and at intermediate frequency in the *L*_*12*_ samples (which is indicative for mutations that occurred in *L*_*1*_ or *L*_*2*_)

The estimation of the mutant frequencies is therefore more accurate on *haplotigs* as the reads of the alleles derived from the homologous chromosome do not dilute the signal as compared to the estimation made with the alignments to *homotigs*. Among the 46 de novo mutations, 29 were found on haplotigs suggesting that all the aligned reads were only derived from one allele. Among those, five (17%) de novo mutations could be observed in only some of the reads suggesting that they were layer-specific. In fact, these layer-specific mutations could even be assigned to an actual layer as they were supported by half of the reads in the *L*_*123*_ samples, while the mutant alleles were virtually absent in the *L*_*12*_ samples suggesting that they occurred in *L*_*3*_ (Fig. 3).

In contrast, the remaining 24 (83%) de novo mutations could be observed in nearly all the reads in both the *L*_*123*_ and the *L*_*12*_ samples suggesting that these mutations were in fact not layer-specific, but that they were present in all layers. This is in agreement with previous observations where meristematic cells can migrate between layers and that layer identity is not absolute [2].

The mutant allele frequencies of the 17 de novo mutations detected on *homotigs* revealed the same types of mutations (with the difference that the mutant allele frequencies were generally only half), with four (23%) observed in *L*_*123*_ only (or the read counts on the mutant allele were negligibly few in *L*_*12*_) indicating they occurred in *L*_*3*_ (Fig. 3).

To understand the layer-specific dynamics of gene conversions, we also analyzed the allele frequencies of the converted alleles. It is important to note that our approach could not reveal gene conversions that removed alleles only from some layers (while the alleles are still present in other layers). Here we could only find gene conversions that removed alleles entirely. We found one predominant type of gene conversions (44 out of 54, 81%) where the allele frequencies of the converted alleles were intermediate in the *L*_*12*_ samples and low in the *L*_*123*_ samples (Fig. 3; Supplementary Table S3). This suggested that the heterozygous alleles were present in *L*_*1*_ or *L*_*2*_ before they got converted (Fig. 3).

In summary, approximately 20% of the de novo mutations occurred in specific meristematic layers only, while 80% were found in cells derived from all layers. In contrast, we found 81% gene conversions were layer-specific, which however is influenced by the limitation that we could only find gene conversions that removed an allele from all cell layers.

### Distribution of somatic mutations across individual branches

Our samples were based on bulked leaves sampled from the upper (mutant) and lower (wildtype) part of the tree (Fig. 1a). To get a better understanding of the distribution of the mutations across the tree, we genotyped the distribution of 29 mutant and wildtypes alleles across the tree (including 20 gain-of-heterozygosity and 9 loss-of-heterozygosity separating the mutant and wildtype parts). The individual leaves that we used for the genotyping were sampled from seven upper and six lower scaffolds of the tree (Fig. 1a; Material and methods). Besides in individual leaves, we further tested the existence of the 29 mutations in additional samples, including two (so-far un-tested) pools of leaf samples and two petiole samples for the mutant part, and similarly for the wildtype part. In total, each mutation was genotyped in 21 DNA samples.

**Fig. 4:**
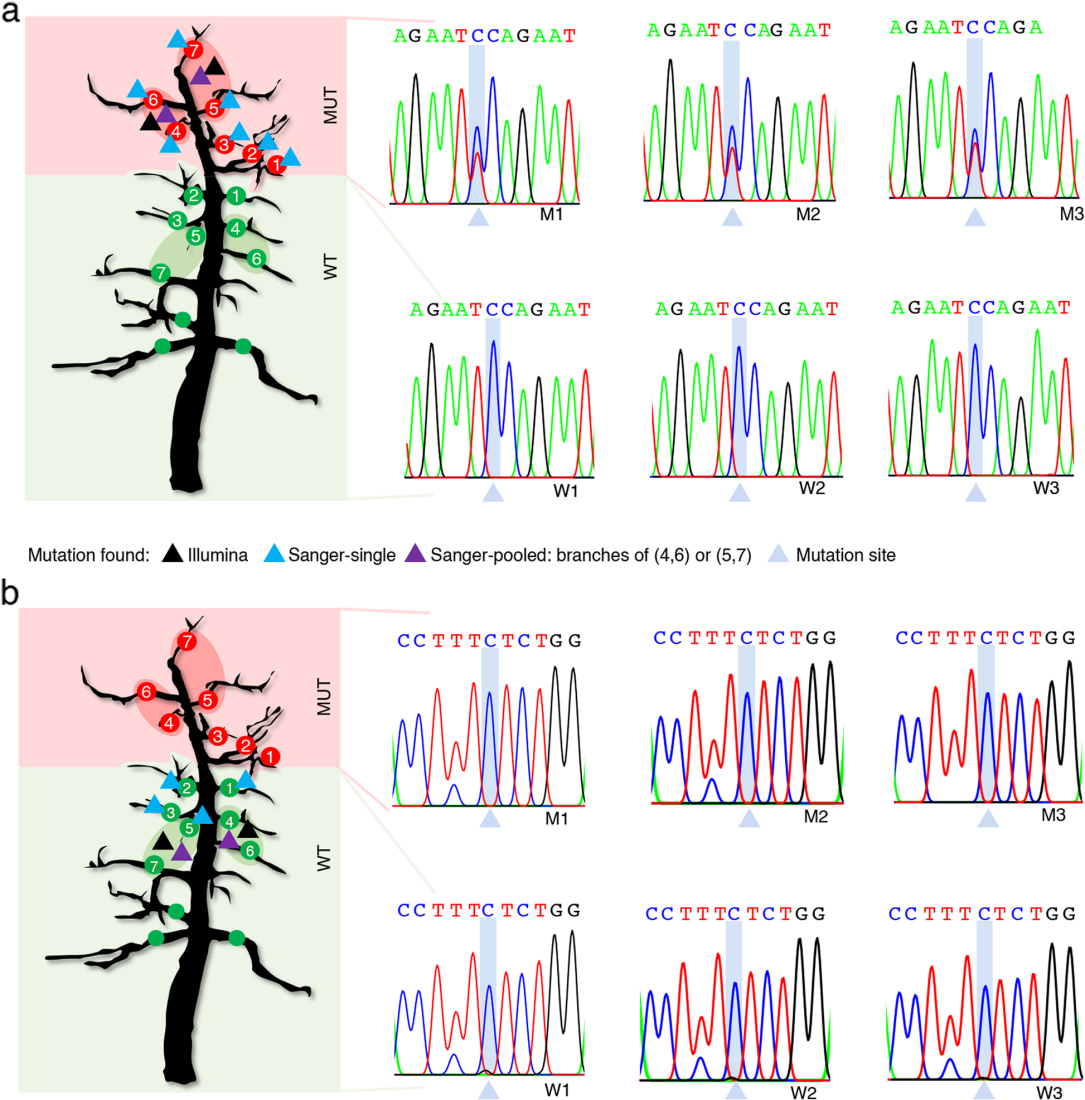
Examples of mutations validated using Sanger sequencing and their distribution across branches. **a** Example of a GOH mutation found in the mutant branches (No. 2 on fl_10473; Supplementary Table S3). MUT: mutant, WT: wildtype. Sanger sequencing showed comparable intensity for both wildtype (‘C’) and mutant alleles (‘T’) at mutation sites at mutant branches, but the signals were not observed at wildtype branches. **b** Example of a LOH mutation found in the wildtype branches (No. 43 on fl_2910; Supplementary Table S3). Sanger sequencing showed weaker intensity for mutant alleles (‘G’) than wildtype alleles (‘C’) at mutation sites at wildtype branches, but the signals were not observed at mutant branches. Full examinations of 29 mutations are shown in Supplementary Figs. 2–30

Among the 20 gain-of-heterozygosity mutations (Fig. 4a; Supplementary Figure S2-21), we found 17 in all seven mutant scaffolds of the tree and not in any of the lower wildtype scaffold samples (Supplementary Figure S2-4,6–7,9–20). Of the remaining three, one was found in six of the seven mutant scaffolds while genotyping of the seventh sample was not successful (Supplementary Figure S5); one was also found in six mutant scaffolds plus one wildtype scaffold (Supplementary Figure S8); while the last one could only be found in pooled samples of the upper mutant scaffolds (Supplementary Figure S21). The nine loss-of-heterozygosity mutations were all found in two to six samples of the lower wildtype part of the tree but not in any of the samples of the upper part of the tree (Fig. 4b; Supplementary Figure S22-30).

Taken together, genotyping of the mutations across the tree confirmed that most of the mutations that were assigned to the mutant scaffolds, were specific to the mutant part of the tree.

### Identification of candidate genes causing enhanced red fruit skin coloration

**Fig. 5:**
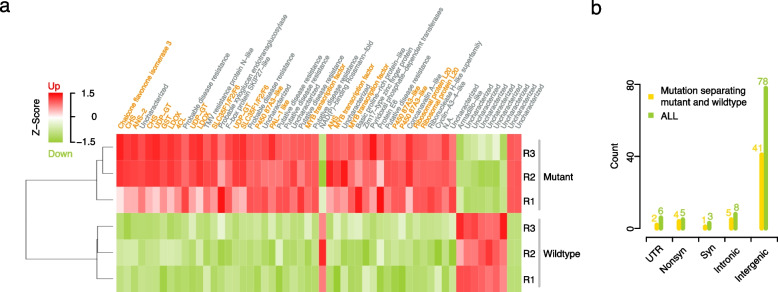
Differential gene expression analysis between mutant and wild-type fruit peel. **a** 56 differentially expressed genes (DEGs) including 48 genes up-regulated and 8 genes down-regulated in the mutant peel. Genes labeled with orange names were involved in flavonoid or anthocyanin biosynthesis and regulation (including MYB transcription factors). **b** Distribution pattern of mutational effects on gene integrity

To understand the molecular basis of the spontaneous switch in fruit coloration, we first analyzed genome-wide expression differences between the skin of mature mutant and wildtype apples. This revealed 56 differentially expressed genes (DEG), including 48 genes, which were up-regulated in the mutant fruits (Fig. 5a; Materials and methods). Apart from putative disease resistance and uncharacterized genes, 18 of the DEGs were involved in flavonoid or anthocyanin biosynthesis and regulation, including the *MdMYB*1 transcription factor *MD09G1278600* that is known to regulate apple skin color [14–18] (Supplementary Table S4; Materials and methods). While this support our assumption that the spontaneous changes introduced changes in the anthocyanin production, we could not find mutations within the DEGs or in their flanking regions (up to hundreds of kb) suggesting that the expression differences are downstream effects of a mutation somewhere else in the genome [31], or that other changes are responsible for the change.

We examined the 53 somatic changes that discriminated the mutant and the wildtype parts for their effects on gene integrity, which were scattered across the genome without any significant clustering regarding GOH and LOH (Fig. 1c). Of those, 12 mutations were located in genes, including four nonsynonymous and one synonymous change within exons, five mutations in introns, and two in UTRs (Fig. 5b; Supplementary Table S3). One of the genes with a nonsynonymous GOH mutation (Fig. 1c; Supplementary Table S5-7) was an F-box gene (at Chr09:32,528,560, when mapped to the GDDH13 v1.1 genome assembly) which resides within a GWAS peak that was recently associated with red skin coloration [32–35] as well as a LOH mutation at Chr09:4,621,163, which was 43 bp away from an anthocyanins 5-aromatic acyltransferase-like protein and near a QTL that was identified as involved red skin coloration variation [34]. Another nonsynonymous mutation was found in a homolog of the *Arabidopsis thaliana HSP90* gene (MD08G1011200), which has been shown to influence morphogenetic responses to environmental stresses such as leaf colors [36].

While it is not possible to prove the causality of a mutation with sequencing data alone, it is possible to rank mutations. Here, we found a small number of mutations and genes, which serve as prime candidates for further analysis to finally reveal the actual reason for the spontaneous fruit coloration switch of RubyMac.

## Discussion

Unlike germline mutations, most of the somatic mutations are not inherited through seeds but can lead to immediate and observable, phenotypic changes. Occasionally, such mutations can result in the development of phenotypic change with significant horticultural value [56, 57]. If such sport mutations can be propagated clonally, they can give rise to new cultivars with desirable traits. Sport mutations have been observed in many fruit trees. For example, a bud sport within the ‘Nanguo’ pear has led to a new cultivar called ‘Nanhong’ (*Pyrus ussuriensis*) [58]. The underlying mutation resulted in higher expression levels of an anthocyanin biosynthesis gene and an anthocyanin transporter gene, which in turn led to the formation of a red fruit skin. Likewise, in grapes, a bud sport in the variety ‘Cabernet Sauvignon’ changed the pale-coloured berries to bronze [59] and independent layer-specific mutations in the ‘Pinot noir’ grape led to the loss in red skin coloration in both ‘Pinot blanc’ and ‘Pinot gris’ and the emergence of two important white wine types [60]. Not only color, but also other traits can be subject of change through various sport mutations. For instance, a bud sport in the peach variety ‘Zhaoyue’ (*Prunus persica*) resulted in a transformed shape turning the fruits from flat to round [61]. In ‘Autumn Gala’ (*Malus domestica*), it was found that a 2.8-Mb hemizygous deletion was causal for the late maturation and the loss in a functional copy of *MdACT7,* which plays an essential role in plant development, was suggested to be responsible for slowing fruit development and delaying fruit maturation [62]. Since this mutation involves a large deletion, it is likely not readily reversible, thereby minimizing the potential for reversion. In another instance of altered fruit maturation, differential expression of a small heat shock protein (*MdHSP17.5*) gene in ‘Fuji’ is suggested to be linked to altered ripening profiles [63]. Other genes like *MdACO1* with potential for influencing maturation date were also identified [64]. But despite their proven value and common use, characterizing somatic mutations on genome scale has not been performed for many important sport mutations in fruit so far. Additional targets in apple would be discovery of the genetic mechanisms for phenotypic differences in tree architecture, early fruit maturation, fruiting spur formation, fruit size, and fruit shape. It is likely that there is more than one genetic route to these and other sport phenotypes. Because of their practical value to the grower in terms of production and sales, it is particularly important to explore the underpinnings of mutations that impact maturation timing and color development [2].

Somatic mutations that occur in the meristem have the chance to be propagated into large sectors and thereby drastically change the behavior of large parts of a plant. Knowing the genetic basis of bud sport mutations can broaden our understanding of phenotypic change and accelerate breeding related efforts, specifically as they could be introduced to other varieties using gene editing. Analyzing the different sectors of an apple tree with a sport mutation that changed fruit coloration, we identified somatic mutations that distinguished the wildtype and mutant genomes and thereby we were able to define candidate mutations that might underly the change in fruit coloration.

Unexpectedly, however, we did not only find gain-of-heterozygosity mutations, but also a similar number of loss-of-heterozygosity mutations. To investigate the general differences between them, we investigated the meristematic layers using tissue-specific DNA samples that were enriched for cells derived from specific layers. Due to our sampling, we could not determine the frequency of L_1_ mutations, which previously have been found to outnumber L_2_ mutations [19, 37]. However, here we also found that *L*_*3*_ specific mutations are also more frequently occurring than mutations in *L*_*2*_. As only mutations in *L*_*2*_ are passed on the next generation, this further points to an increased genome stability in L_2_ as compared to the other layers.

Moreover, somatic de novo mutations were enriched for GC =  > AT transitions, which is consistent with the spectrum of germline mutations in plants [29], while loss-of-heterozygosity mutations were biased towards AT =  > GC transitions. This suggested that the genome-wide GC-content might be controlled by both novel mutations and the mismatch repairing systems that introduce gene conversions, which can counteract each other’s biases. It should be noted, we cannot fully exclude the possibility that the loss-of-heterozygosity mutations were in fact de novo mutations that occurred only in the lower part of the tree. But if we assume that the gene conversions were wrongly assigned (and were in fact de novo mutations), we would expect them in similar layers as all other de novo mutations, which, however, was not the case (gene conversions were specific to *L*_*1*_ or *L*_*2*_ while most of the layers-specific mutations were found in *L*_*3*_). This suggests that the gene conversions were indeed no new mutations – even though a recent analysis of an apricot tree hardly showed any gene conversions [19]. Another note of caution, in our interpretation we have assumed a three-layer model of the meristem, as well as the persistency of the layer identify in somatic tissue. While both might be a simplification with respect to the apple tree analyzed here, our analysis does confirm that layer-specificity in general needs to be considered when analyzing somatic mutations in plants and that the search for mutations that are fixed in one haplotype would have missed some of the somatic mutations.

Future application of single cell sequencing technologies might help to overcome some of the challenges presented and help to identify differences in the mutational profiles of different layers and thereby help deepen our understanding of the dynamics of somatic mutations and their inheritance in long-lived plants.

In addition, while genetic mutations and natural selection are primary drivers of trait changes, epigenetic mechanisms provide a layer of control over how genes are expressed without altering the underlying DNA sequence. These modifications, such as DNA methylation, histone modifications, or non-coding RNA regulation, are often triggered by environmental stress, meaning that two genetically identical plants can exhibit different traits if exposed to varying environmental conditions. This epigenetic plasticity enables plants to adapt to environmental challenges rapidly, resulting in phenotypic diversity [65, 66]. For instance, by analyzing a 10-year-old apple tree of ‘Oregon Spur II’ with multi-omics sequencings, hypermethylation in the promoter region of *MdMYB10* was found to be responsible for inducing a suppression in red fruit coloration^67^. Thus, in addition to sequencing the genome to identify any genetic mutations, conducting epigenetic analysis helps uncover potential regulatory changes. This allows for a better understanding of whether the phenotypic change is due to genetic mutations or epigenetic modifications, which can both play roles in the development of sport mutants.

## Conclusion

Our analysis revealed that somatic mutations in the *RubyMac* apple tree occurred with clear layer-specificity, where the gene conversions mainly being GC-biased counteracted de novo mutations tending mainly being AT-biased. In addition, we identified somatic mutations which distinguished the wildtype and mutant genomes, leading to candidate mutations that might underly the change in fruit coloration. Our study has provided novel insights into mutations underlying bud sports of fruit trees and highlighted the importance of analyzing somatic mutations at cell layer resolution.

## Materials and methods

### DNA/RNA extraction and sequencing library preparation

All experiments were performed with the permission of the relevant institutions. Trichome tissue (*L*_*1*_) was separated by freezing mutant and wildtype leaves of the apple tree* (Malus domestica cultivar McIntosh RubyMac)* in liquid nitrogen and dislodging the trichomes into a 50-mL Falcon tube using a soft paintbrush. The liquid nitrogen was allowed to boil off leaving the trichomes behind. Leaf surface tissue (*L*_*12*_) was isolated from mutant and wildtype leaf petioles by scraping the petiole with a razor blade to remove the outer tissue layers and directly drop them into liquid nitrogen and stored at -80 °C until use. Whole leaves (*L*_*123*_) as well *L*_*12*_ tissue was ground in liquid nitrogen in a mortar and pestle prior to extraction. DNA was extracted using the DNeasy Plant Mini Kit (Qiagen, Hilden, Germany). DNA samples of whole leaves (*L*_*123*_) were used for both Illumina and Sanger sequencing.

Fruit skin from the mutant fruit and wildtype fruit (each with three replicates) was respectively obtained using a handheld fruit peeler to separate the skin (as well as a few millimeters of cortex tissue) from the fruit. Tissue was ground in liquid nitrogen using a mortar and pestle and stored at -80 °C until use. Total RNA was extracted from according to Gasic et al. [38] and adapted for microcentrifuge tubes by using 1/10 the recommended volumes of all solvents and sample weights. Following the initial extraction, the extracted RNA was purified using the RNeasy mini-spin column protocol (Qiagen, Hilden, Germany).

All sequencing libraries were prepared at Max Planck-Genome-center Cologne, Cologne, Germany. Twelve DNA libraries were sequenced using Illumina HiSeq2500/MiSeq platforms under 250 ~ 300 bp paired-end reads mode. Six RNA libraries (mutant with three replicates and wildtype with three replicates) were sequenced using Illumina HiSeq3000 under 150 bp single-end reads mode.

### Genome size estimation

For each of the eight *L*_*12*_ and *L*_*123*_ samples, *k*-mer counting (*k* = 21) in the respective short reads was performed using the tool *jellyfish* [39]. Given the *k*-mer frequency histograms, genome size and rate of heterozygosity were estimated using *findGSE* [40] under heterozygous mode. Averages of the eight estimations (with standard variations) were defined as the final value.

### Genome assembly and annotation

Genome assembly was performed using *DiscovarDeNovo* [21] with default settings, which led to the *RubyMac* genome assembly of 875 Mb including all ≥ 1 kb contigs. Note, the assembly size was larger than the haploid genome size of 731 Mb as estimated by *findGSE* [40] and also larger than the reference assemblies of *Golden Delicious* [26, 41], due to the high heterozygosity of *RubyMac* (1.2 ± 0.1% estimated by *findGSE*), which resulted in haplotype-specific contigs (or *haplotigs*). Using a computational pipeline to identify haplotigs [42], we further characterized haplotigs with a combined length of ~ 242 Mb within the *RubyMac* assembly explaining the inflated assembly size (method give below). For gene prediction, Illumina single-end 150 bp RNA reads were aligned to the assembly using *tophat2* [22] with options -N (read mismatches) and –read-edit-dist as 10, –library-type fr-firststrand and the others as defaults. The result BAM file was provided to *AUGUSTUS* [23] *bam2hints* to generate a GFF file for guiding gene annotation by *AUGUSTUS* with options –species = Arabidopsis –extrinsicCfgFile = /augustus-master/config/extrinsic/extrinsic.cfg –alternative-from-evidence = true –UTR = on –progress = true –allow_hinted_slicesites = atac –uniqueGeneId = true.

### Evaluation of assembly

The assembly was evaluated using the BUSCO analysis at both annotation and sequence level. For this, the *busco* tool (version 5.7.1) [43] and the lineage dataset for plants (https://busco-data.ezlab.org/v5/data/lineages/embryophyta_odb10.2024-01-08.tar.gz) were used (total BUSCO groups 1614), with options “-i genome.fasta -l embryophyta_odb10 -o *RubyMac*_genome -m genome” and “-i protein.fasta -l embryophyta_odb10 -o R*ubyMac*_prot -m prot”. Also, completeness of the assembly was evaluated with *Merqury* version 1.3 [44] with default parameters.

Scaffolding of the *RubyMac* contigs using the GDDH13 v1.1 reference genome was performed using the *ragtag scaffold* function with default parameters (*ragtag* version: v2.1.0 [45]): ragtag.py scaffold ref query, where ref refers to the fasta file of GDDH13 v1.1 and query refers to the fasta file of *RubyMac*.

### Haplotig identification

To guarantee a sufficient sequencing depth required by this analysis, paired-end reads from two wildtype *L*_*123*_ samples were merged as one set, each contributing ~ 75x (Supplementary Table S1). Combined reads were aligned to the *RubyMac* assembly using *minimap2* [46], and the bam file was sorted and indexed using *samtools* [47]. Then “*purge_haplotigs readhist*” was applied to generate a sequencing depth histogram for each contig. Expecting ~ 150 × sequencing depth for a homotig and ~ 75 × for a haplotig, we set 20, 122, and 300 as depth cutoffs required by *Purgen_Haplotigs*, corresponding to low (“valley” on the left of the heterozygous peak in the depth histogram), middle (“valley” between the heterozygous and homozygous peaks) and high (nearly two times of the homotig sequencing depth). With these cutoffs, “*purge_haplotigs contigcov*” would predict a contig as a haplotig if more than 30% of its positions were not covered by 122 ~ 300x, for which “*purge_haplotigs purge*” would search for a matching haplotig and if 85% of the contig can be aligned with others, it would be determined as a haplotig. This analysis led to 59,293 curated primary contigs (~ 630.7 Mb), 44,305 haplotigs (~ 242.2 Mb) and 795 artificial contigs (~ 1.8 Mb).

### Small-scale variation identification and validation with Sanger sequencing

We first identified small-scale variations with a customized pipeline using the *RubyMac* assembly as reference. Specifically, reads of each of the 12 DNA samples were respectively aligned to the reference genome using *bowtie2* [48], and resulted SAMs was converted to BAMs and indexed using *samtools* [47]. With each BAM, read counts were collected for all bases (including deletions) at each position of the reference genome by “*shore consensus*” [49], which simultaneously predicted variations with allele frequency, read coverage and base quality score (maximum 40). Then a somatic mutation would be called at a position, if there was an alternative allele not found in all eight *L*_*12*_ and *L*_*123*_ samples (note that due to the limited sequencing depth of *L*_*1*_ samples, they were not considered in this step).

With this, we selected the initial list of 69 mutations for Sanger sequencing validation, where the alternative allele must be covered by at least 15 reads in at least one sample and not common between wildtype and mutant branches of the tree. The initial list of 69 mutant- or wildtype-specific mutations were genotyped in DNA of individual leaves from seven mutant scaffolds on the tree, two pooled leaf blade samples (mutant scaffolds {5, 7} and {4, 6}) and two pooled petiole samples (mutant scaffolds {5, 7} and {4, 6}), and similarly in DNA of individual leaves from six wildtype scaffolds on the tree, two pooled leaf blade samples (wildtype scaffolds {5, 7} and {4, 6}) and two pooled petiole samples (wildtype scaffolds {5, 7} and {4, 6}), with 19–29 bp primers around the mutations designed by *Primer3* [50] (Fig. 1a; Supplementary Table S3). Generally, each mutation was genotyped in 21 DNA samples. The R package ‘*sangerseqR*’ was used to analyze the Sanger-seq data with the threshold of 0.01 for function *makeBaseCalls* to call (low-frequency) alternative alleles [51].

The initial set of 69 candidate mutations consisted of three subsets, as defined based on three different quality metrices. The first set consisted of 29 mutations, which were called in more than two samples with an alternative base quality above 30 in at least two of the samples. Of those we could confirm 27 mutations (Supplementary Fig. 2–30). The second set consisted of 24 mutations, which were also predicted in more than two samples but with less than two samples with an alternative base quality above 30. Of those, only two were confirmed by Sanger sequencing. The third set, least stringent, included six mutations, which were predicted in only up to two samples with at least one sample with an alternative base quality above 30. None of them could be confirmed by Sanger sequencing. For the remaining 10 cases the PCR sequencing did not yield conclusive results.

While it was obvious that the criteria for the second set were not sufficient to overcome a high rate of false positive, we found that among the eight unconfirmed cases in the first and third subset there were five low-frequency mutations, which potentially could have even been missed with Sanger sequencing. For example, one of those mutations (a loss-of-heterozygosity mutation) was found with a minor allele frequency of less than 0.1 in the whole-genome sequencing data (Supplementary Fig. 31) and was not confirmed by Sanger sequencing. However, within RNA-seq data that we generated from the peels of fruits, all three wildtype replicates again showed 4 ~ 6% reads (read depths: 595-784x) with the alternate allele, while not a single such read showed up in any of the three mutant replicates (read depths: 660-861x; Materials and methods; Supplementary Fig. 31). The identification of the alternate allele in RNA-seq suggested that this was a real difference between the mutant and wildtype branches despite it was missed in the Sanger sequencing.

Taken together, the high false positive rate in second set indicated that mutations with comparably low alternative base quality can usually not be trusted in our dataset. Among the other mutations in other two subsets, we were able to confirm 28 cases with either Sanger or RNA-seq data suggesting the minimum requirement on the base quality that we need to apply to guarantee satisfactory accuracy in predicting mutations, while some of the low-frequency candidate mutations might have been missed in the Sanger sequencing.

Therefore, in the second round of mutation identification, the following criteria were applied in each somatic mutation calling, including minimum alternative allele frequency of 0.1, read coverage of 10 ~ 150x, minimum alternative base quality of 30 (or Q30. Note, if there were > 2 samples with the alternative allele, a mutation was kept only if at least two samples showing Q30. Otherwise, a mutation was kept if at least one sample showing Q30, and both the maximum average numbers of unknown base *N* and mismatches in the read alignments covering the mutation position were set as 5 (corresponding to ~ 2% of the read length of 250 ~ 300 bp). The detected variations were further filtered manually by checking alignments by browsing with IGV [52]. This led to the list of 100 mutations.

In addition, the reported variations (Supplementary Table S3) could also be detected by other tools such as reference-based *MuTect* (version 1.1.4) [24] with options *“–input_file:normal sample1.bam –input_file:tumor sample2.bam*”, where sample1.bam and sample2.bam are BAM files as mentioned above, and reference-free *discosnp* +  + (version 2.2.X) [25] with options “*-r readset.txt -k 41 -b 1*”, where the file readset.txt included the list of fastq files of any pair of samples to compare.

### Definition of gain-of-heterozygosity and loss-of-heterozygosity mutations

We defined a mutation as a gain-of-heterozygosity mutation if it was covered by more than 15 reads in at least one sample (which could be either mutant or wildtype) and at least three wildtype samples did not support the alternative allele with more than two reads. In contrast, a mutation was defined as loss-of-heterozygosity if the alternative allele was found in at least three wildtype samples with a coverage of over 15 while all four mutant samples did not feature more than 3 × of the mutant allele.

### Somatic mutation clustering

Let *r*_*123*_ and *a*_*123*_ respectively represent for the counts of reads carrying reference and alternative alleles for *L*_*123*_ (i.e., blade sample) while *r*_*12*_ and *a*_*12*_ for *L*_*12*_ (i.e., surface sample) in either mutant or wildtype, depending on which one has the alternative allele. The difference between (*r*_*123*_, *a*_*123*_) and (*r*_*12*_, *a*_*12*_) was examined by a two-sided *Fisher*’s exact test (*R* package “stats”). To reduce the effect of randomness in read counts, any value in the set of (*r*_*123*_, *a*_*123*_, *r*_*12*_, *a*_*12*_) for one mutation was taken as the summation of read counts from two replicates. If the test for a mutation gives a *p*-value smaller than 0.05, it was defined as with significant difference in allele frequencies (AFs) of *L*_*123*_ and *L*_*12*_, indicating there was a gain (or loss) of the alternative allele in a layer. This led to three clusters of mutations (on haplotigs and homotigs), a) AF in *L*_*123*_ is comparable to *L*_*12*_, b) AF in *L*_*123*_ is significantly larger than *L*_*12*_, and c) AF in *L*_*12*_ is significantly larger than *L*_*123*_.

### Differential gene expression analysis

The RNA-seq reads of three mutant-related replicates (upper part of the tree) and three wildtype-related replicates (lower part of the tree) were respectively aligned to the 875 Mb *RubyMac* genome using *tophat2* [22], with options “-p 10 -a 10 -g 10 –library-type fr-firststrand” for controlling the number of threads, minimum anchored bases and maximum multiple hits of reads. The BAM files were indexed with *samtools* [47]. Read counts for genes in GFF (generated by *AUGUSTUS* [23]) were extracted with *HTSeq* [53] for each sample. Differential expression (DE) analysis was performed with an *R* pipeline [54], with adjusted *p*-value < 0.05 for considering differential expression. Specifically, read count per gene was extracted using *HTseq.scripts.count*, from which only genes that were covered by a minimum of 10 reads in at least one sample were kept for DE analysis. Using the libraries *limma* and *edgeR* in *R*, the read counts were read in with *readDGE* function. The read counts of six samples were grouped as “MUT” or “WT” according to the phenotype of the branches. Information on genes were organized as “entry-ID”, “Contig”, and “gene-ID”, and duplicated gene identifiers were removed. Then the information on read counts and genes were merged according to gene identifiers. To account for differences in library size, CPM (count per million) values of each gene was calculated with *cpm* function in *edgeR* (formula: *count at gene i in sample j* / *sum(counts at all genes in sample j)* * 1000,000). Then, only genes with CPM values larger than 1.5 in at least three samples were kept for subsequently analysis. To avoid external factors that were not of biological interest, gene expression distributions were normalized with *calcNormFactors* function. Contrasts for pairwise comparisons between samples were set up in *limma* using the *makeContrasts* function. Linear modelling in *limma* was carried out using the *lmFit* and *contrasts.fit* functions. Empirical Bayes moderation was carried out by the *eBayes* function. Significance was defined using an adjusted *p*-value cutoff that was set at 5% by default. For a stricter definition on significance, a minimum of log-fold-changes of 0.6 was simultaneously applied to determine the final list of DE genes.

We also repeated the analysis with the 807 Mb GDDH13 v1.1 reference genome and annotation of *Golden Delicious* [26]. Four DE genes as identified with the *RubyMac* assembly (for which blasts to the NCBI nucleotide databases hit genes of probable copper-transporting ATPase *HMA5*, not available, F-box protein *SKIP27*-like and myosin heavy chain clone 203-like) were not in *Golden Delicious* gene annotation. To be comprehensive, we created a final list of 56 DE genes by merging two sets (Supplementary Table S4).

### Large indels and copy number variation (CNV) identification

The ~ 875 Mb *RubyMac* assembly was indexed with *bowite2* [48]. Reads were aligned for each of the eight *L*_*12*_ and *L*_*123*_ samples with *bowite2* [48], and the BAM files were indexed with *samtools* [47]. Then the BAM files were provided to *CNVnator* (version) [27] for CNV detection with various bin sizes of 50, 200 and 300 bp. After filtering, we could find 199 mutant-specific and 169 wildtype-specific candidate CNVs. However, further checking of the candidates on alignments in IGV did not reveal any convincing ones.

## Supplementary Information

Supplementary Material 1.

Supplementary Material 2.

## Data Availability

Read data of all 12 DNA sequencing libraries, 6 RNA sequencing libraries, and Sanger sequencings of 69 mutations within 21 samples are available under BioProject PRJNA881844 at NCBI. Genome assembly and annotation of ‘RubyMac’ are available at Zenodo [55].

## Abbreviations

AF: Allele Frequency
CNV: Copy Number Variation
DEG: Differentially Expressed Gene
GWAS: Genome-Wide Association Study
GOH: Gain of Heterozygosity
LOH: Loss of Heterozygosity
SV: Structural Variation
